# The comparison of polymorphism among *Avena* species revealed by retrotransposon-based DNA markers and soluble carbohydrates in seeds

**DOI:** 10.1007/s13353-023-00748-w

**Published:** 2023-01-31

**Authors:** Piotr Androsiuk, Sylwia Eryka Milarska, Justyna Dulska, Wioleta Kellmann-Sopyła, Joanna Szablińska-Piernik, Lesław Bernard Lahuta

**Affiliations:** Department of Plant Physiology, Genetics and Biotechnology, Faculty of Biology and Biotechnology, University of Warmia and Mazury in Olsztyn, ul. Oczapowskiego 1A, 10-719 Olsztyn, Poland

**Keywords:** Molecular markers, iPBS, Genetic diversity, Gas chromatography, Cereal seeds

## Abstract

Here, we compared the polymorphism among 13 *Avena* species revealed by the iPBS markers and soluble carbohydrate profiles in seeds. The application of seven iPBS markers generated 83 bands, out of which 20.5% were polymorphic. No species-specific bands were scored. Shannon’s information index (I) and expected heterozygosity (H_e_) revealed low genetic diversity, with the highest values observed for *A. nuda* (*I* = 0.099; *He* = 0.068). UPGMA clustering of studied *Avena* accessions and PCoA results showed that the polyploidy level is the main grouping criterion. High-resolution gas chromatography revealed that the studied *Avena* accessions share the same composition of soluble carbohydrates, but significant differences in the content of total (5.30–22.38 mg g^−1^ of dry weight) and particular sugars among studied samples were observed. Sucrose appeared as the most abundant sugar (mean 61.52% of total soluble carbohydrates), followed by raffinose family oligosaccharides (31.23%), *myo*-inositol and its galactosides (6.16%), and monosaccharides (1.09%). The pattern of interspecific variation in soluble carbohydrates, showed by PCA, was convergent to that revealed by iPBS markers. Thus, both methods appeared as a source of valuable data useful in the characterization of *Avena* resources or in the discussion on the evolution of this genus.

**Supplementary Information:**

The online version contains supplementary material available at 10.1007/s13353-023-00748-w.

## Introduction

The genus *Avena* L. (Poaceae), which can be found within the Aveneae tribe, is represented by about 30 species (Baum 1977). The majority of oats represent wild and weedy species, but there are also crop species among them—*Avena strigosa* Schreb., *A. abyssinica* Hochst., *A. byzantina* C.K., and *A. sativa* L. *A. sativa* is the most commonly cultivated oat used for animal feed, human consumption, and industry purposes (Boczkowska and Tarczyk 2013). All representatives of the genus *Avena* are self-pollinated annuals, with the exception of *A. macrostachya* which is an outbreeding perennial (Katsiotis et al. 1997). Genus *Avena* contains diploid (AA and CC), tetraploid (AACC and AABB), and hexaploid (AACCDD) species with a basic chromosome number of seven (Baum 1977, Loskutov and Rines 2011). Surprisingly, there is no information about diploid species with BB or DD genome, although *A. canariensis* was postulated as a candidate DD genome (Yan et al. 2016). Cytogenetic analyses revealed that the D genome and B genome are highly similar to the A genome type (Rajharthy and Thomas 1974; Katsiotis et al. 1997; Tomaszewska et al. 2022). Detailed cytological studies showed that species within the genus *Avena* share their genome(s) with one another, which proves their evolutionary relationships (Linares et al. 1998). Moreover, the evolution of the oat nuclear genomes appeared as a complex process which involved divergence at the diploid level from a common diploid ancestor and then convergence, followed by divergence, at the polyploid level (Thomas 1992).

Oat grains are known for their high level of calcium, β-glucan soluble fiber (Andon and Anderson 2008; Jenkins et al. 2002; Yarnell and Abascal 2001a, 2001b), and high-quality oil and protein content (Fardet 2010; Peterson 2001). Furthermore, they are rich in antioxidants as well as anti-inflammatory and antiatherogenic compounds (Daou and Zhang 2012). As a result, oats have attracted the attention of breeders. However, despite intensive efforts which have been made to describe the genetic diversity and perform biochemical characteristics of particular collections, representing existing gene pools of cultivated oats, we are still lacking their comprehensive characterization. Currently, available literature shows that many wild and weedy *Avena* species can be used in breeding programs as donors of many valuable traits which enable the increase of the yield and grain quality (e.g., *A. fatua*, *A. sterilis*, *A. ludovicina*, *A. occidentalis*; Trofimovskaya et al., 1976; Frey 1991; Miller et al. 1993; Loskutov et al. 2021) as well as protein content in straw and green matter (e.g., *A. abyssinica*, *A. magna*, *A. ludovicina*, *A. sterilis*; Mal 1987; Rezai 1977; Loskutov and Rines 2011; Ociepa 2019). Wild *Avena* species can be used also to improve the cultivated forms of oat in terms of disease and pathogen resistance, earliness, or highly productive tillering (Marshal and Shaner 1992; Leonard et al. 2004; Okoń et al. 2016, 2018; Gordon et al. 2022). The lack of precise characterization of the genetic resources is the main obstacle to successful and efficient breeding programs. The knowledge about the genetic diversity presented in cultivated oats and their wild relatives, information about relationships among these species, and their detailed biochemical characteristic is crucial not only for utilization of the rich genetic diversity of wild *Avena* species (Govindaraj et al. 2015) but also for conservation of this valuable genetic resources (Leggett 1996).

One of the special characteristic features of the eucaryotic genomes is the presence of repetitive elements which are present in a high number of copies scattered throughout the genome. These sequences can be identified as tandemly repeated elements (e.g., microsatellites) or transposable elements (TEs). The discovery of the TEs change our way of thinking about the mechanisms responsible for genome diversification and evolution (McClintock 1984; Kunze et al. 1997). TEs, due to their ability to replication and change their location in the genome, can induce various types of mutations beginning with insertion to protein-coding genes, illegitimate recombinations, and genomic rearrangements, up to chromosomal breakage (Feschotte et al. 2002; Feschotte and Pritham 2007; Wessler 2006; Almojil et al. 2021). The TEs activity has also an influence on gene expression as their insertion within the sequence of the particular gene not only may destroy the gene structure and prevent its transcription but also may alter the gene expression profile by insertion into its promoter region (Kumar and Bennetzen 1999; Levin and Moran 2011; Bourque et al. 2018). Based on the structure and mechanisms of transposition, TE is divided into two classes: class I which contains retrotransposons (retro-TEs) and class II which includes DNA transposons (DNA-TEs). Retrotransposons, which are “copy and paste” elements, appear as a very important fraction of the higher-plant genomes. For example, in maize (*Zea mays*), TEs constitute for 84.2% of its genome (including 75.6% of retro-TEs and 8.6% of DNA-TEs), in wheat (*Triticum aestivum*) for 79.8% of the genome (63.7% of retro-TEs and 14.9% of DNA-TEs), whereas in tomato (*Solanum lycopersicum*) for 63.2% of the whole genome (62.3% of retro-TEs and 0.9% of DNA-TEs) (Bonchev 2016). As a consequence retrotransposons activity is presented as one of the major factors responsible for the expansion of plant genomes (Kumar and Bennetzen 1999).

Although there were a number of studies on genetic diversity and differentiation as well as the evolution of species representing genus *Avena*, TEs were poorly studied so far in this group of plants. However, based on the analyses of available sequencing data, it is known that repetitive elements (LTR retrotransposons make up most of the classified elements) may comprise even 83% of the *Avena* genomes (Maughan et al. 2019). Moreover, few studies can be found which were devoted to the identification and characterization of TEs in *Avena strigosa*, *A. sativa*, *A. sterilis*, *A. vaviloviana*, *A clauda*, and *A. magna* (Tomás et al. 2016; Linares et al. 1999; Katsiotis et al. 1996). Retrotransposons have proved their high suitability for the development of many DNA marker systems like SSAP (Waugh et al. 1997), RBIP (Flavell et al. 1998), IRAP, and REMAP (Kalendar et al. 1999) or iPBS (Kalendar et al. 2010), which appeared as highly effective molecular tools with a number of applications in plant genetics and breeding (Kalendar et al. 2018). Unfortunately, their application in genetic studies on *Avena* species is very limited, with few papers reporting the application of REMAP for comparative analysis of selected *Avena* species (Tomás et al. 2016; Paczos-Grzęda and Bednarek 2014) or joint AFLP and SSAP markers application for construction of a genetic map of diploid *Avena* (Yu and Wise 2000).

The main limitation of all DNA marker techniques which are based on retrotransposons is the need for sequence information to develop specific PCR primers, allowing the amplification of unique, retrotransposon-related fragments. This fact hampers the exploitation of the above-mentioned molecular techniques by making it available generally for the model organisms or utilized crops for which genomic data are available. Fortunately, a new retrotransposon-based DNA technique was developed, called iPBS (Kalendar et al. 2010), which exploited the conserved region of the primer binding site (PBS) of LTR retrotransposons, which is directly involved in the initiation of the reverse transcription during the replication cycle of retrotransposon. In contrast to other retrotransposon-based molecular markers that require specific primers for the flanking regions of the particular retrotransposon, iPBS uses universal primers which enable the amplification of various types of LTRs. So far, there are several studies in which dominant iPBS markers were successfully applied for the estimation of genetic diversity and differentiation of plants (Bonchev and Vassilevska-Ivanova 2020; Khapilina et al. 2021; Naeem et al. 2021) and fungi (Ates et al. 2019; Turzhanova et al. 2020; Erper et al. 2021). According to our knowledge, iPBS markers have only been used once for the genetic study of genus *Avena*, reporting genetic diversity and differentiation of Latvian *A. fatua* populations (Nečajeva et al. 2021).

In the present study, retrotransposon-based iPBS markers were used for the first time to assess DNA polymorphism and genetic relationships between 13 *Avena* species. In our research, four levels of organization of studied material were applied to establish mutual relationships between studied *Avena* accessions: species level, the taxonomic system of species within genus *Avena* according to Baum (1977), ploidy, and genome type. Biochemical characteristics of the seeds, for example, the composition and content of soluble carbohydrates (mainly raffinose family oligosaccharides, RFOs), are also commonly used for the estimation of intra- and inter-specific diversity. The suitability of that approach was demonstrated for the species representing the genus *Lupinus* (Piotrowicz-Cieslak 2005), *Vicia* (Lahuta et al. 2018; Lahuta et al. 2020), *Lathyrus* (Ibrahim et al. 2021), *Agave*, *Dasylirion* (Mancilla-Margalli and Lopez 2006), and for *Vitis vinifera* L. varieties (Bhouri et al. 2016) or willow cultivars (Budny et al. 2021). Oat grains are also abundant in sugars and oligosaccharides (MacLeod and McCorquodale 1958; Kaur et al. 2019). Therefore, we made an attempt to determine the usefulness of intra- and inter-specific variation in soluble carbohydrate profiles in caryopses for the characterization of the 13 *Avena* species. In our project, we decided to combine both approaches to characterize the selected *Avena* accessions and to compare the revealed polymorphism

## Materials and methods

### Material

The research material consisted of caryopses of 13 species of the genus *Avena*, represented by 1–9 different, randomly selected accessions, that we were able to obtain from the National Centre for Plant Genetic Resources (NCPGR) of the Plant Breeding and Acclimatization Institute in Radzików, Poland; Nordic Gene Bank (NGB) and N.I. Vavilov All-Russian Institute of Plant Genetic Resources (VIR). The list of all 60 tested *Avena* accessions is provided in Table S1.

### DNA extraction and iPBS genotyping

DNA extraction was performed with the use of the CTAB method by Murray and Thompson (1980) with some modifications (Polok, 2007). For DNA extraction, pooled (bulked) samples were applied in which particular *Avena* accession was represented by an equal amount of fresh tissue collected from 15 randomly selected individuals grown from seeds in a greenhouse of the Department of Plant Physiology, Genetics and Biotechnology at the University of Warmia and Mazury in Olsztyn, Poland. Young, healthy leaves were collected, cleaned, and stored at −20 °C until DNA extraction. DNA quality was verified by agarose gel electrophoresis, and its concentration was determined spectrophotometrically using NanoDrop (ND-1000 UV/Vis).

Based on the results of initial tests of 34 PBS primers (Kalendar et al. 2010), a set of seven primers that gave polymorphic, clearly identifiable, and repeatable bands were selected for further analyses (Table 1). The initial tests and optimalisation of PCR conditions for studied *Avena* species were conducted according to the procedure described in Milarska et al. (2022). The reproducibility of band profiles for each of the selected PBS primers was verified. This verification was based on a comparison of the electrophoretic profiles for selected eleven *Avena* accessions. Data were generated and compared in two replicates. Gels were then checked to identify iPBS amplicons (bands) in one or both replicates. Amplification was performed according to the procedure described by Kalendar et al. (2010) with modifications (Androsiuk et al. 2015). The PCR products were then analyzed by horizontal electrophoresis (1 × TBE buffer at 100 V for 2 h) in 1.5% agarose gel stained with 0.5 mg/ml ethidium bromide.

**Table 1:** Summary on the PBS primers used in the study.

Primer	Sequence	Tm [°C]	Total number of bands [N_B_]	Polymorphic bands [P]	PIC
2076	CTCATGATGCCA	54	9	2	0.10
2085	ATGCCGATACCA	50	10	2	0.08
2224	ATCCTGGCAATGGAACCA	50	12	3	0.12
2229	CGACCTGTTCTGATACCA	52	12	1	0.04
2253	TCGAGGCTCTAGATACCA	58	12	3	0.07
2376	TAGATGGCACCA	56	7	3	0.21
2378	GGTCCTCATCCA	56	21	3	0.06
Average			11.85	2.4	0.10
Sum			83	17 (20, 5%)	–

### Genetic diversity analyses

All bands that could be reliably read across all *Avena* accessions were scored as either present (1) or absent (0) and treated as single dominant loci. The obtained binary matrix (Table S2) was subjected to further analyses and the following parameters were estimated: total number of bands per primer (N_B_), percentage of polymorphic bands (P), Shannon’s information index (I), expected heterozygosity (H_e_), genetic similarity for each pair of accessions according to Dice (1945), as well as standard Nei’s genetic distance and identity (Nei 1972; Nei 1978) for each pair of analyzed species or genome types. Dice similarity values were then used for grouping analysis of *Avena* accessions using UPGMA clustering implemented in PAST 3.18 (Hammer et al. 2001). The matrix containing Nei’s genetic distance values was used to perform the principal coordinate analysis (PCoA) in GenAlEx 6.5 (Peakall and Smouse 2006, 2012) to assess the genetic associations of the *Avena* species and also for UPGMA clustering to estimate relationships between *Avena* genome types. Moreover, the value of polymorphic information content (PIC) for each marker was estimated using the formula described by Roldan-Ruiz et al. (2000).

Finally, the obtained data was also subjected to molecular variance analysis (AMOVA). Analysis of molecular variance was performed with Arlequin 3.5. For this analysis, the iPBS data was treated as haplotypic, comprising a combination of alleles at one or several loci (Excoffier et al. 2005). The significance of the fixation indices was tested using a non-parametric permutation approach, the method implemented in Arlequin 3.5 (Excoffier et al. 1992; Excoffier et al. 2005). The analysis included the partition of the total iPBS variation between *Avena* species not only into within- and among-species variation components, but also with an application of additional structuring of the data which encompassed *Avena* sections according to Baum (1977), ploidy, and genome types. Furthermore, average gene diversity over loci (π_*n*_) was also estimated for specific members of these four groups, according to Tajima (1983) and Nei (1987) model implemented in Arlequin 3.5.

### Analysis of the soluble carbohydrates

The analysis of the soluble carbohydrates profiles was carried out on a group of 47 selected accessions representing 13 species of the genus *Avena* (Table S1). The research material consisted of ripe, husked *Avena* caryopses, which were grounded in a ball mill homogenizer (22 Hz for 2 min). Samples with a weight of 40 mg were prepared in three replicates for each accession. Extraction of carbohydrates was carried out in a mixture of ethanol and water (1: 1, v/v), containing xylitol as an internal standard, at 90 °C for 30 min. The qualitative and quantitative analyses of the soluble carbohydrates were performed using the high-resolution gas chromatography method, as described earlier (Lahuta 2006).

The obtained data were statistically analyzed with the use of Statistica v.12 software (StatSoft Inc., Tulsa, OK, USA). The significance of differences between mean values was determined by a one-way analysis of variance. A post-hoc analysis using Fisher’s least significant difference (LSD) test was carried out to compare the means. Differences were considered to be significant when *p* < 0.05. Moreover, principal component analysis (PCA) was used to visualize the structure of relationships between the studied *Avena* species. PCA included eight variables (fructose, glucose, sucrose, raffinose, stachyose, *myo*-inositol, galactinol, and di-galactosyl *myo*-inositol).

## Results

### Genetic variation

Genetic analysis of 60 *Avena* accessions with the use of seven PBS primers identified 83 amplification products (bands). The highest number of bands (21) was revealed by PBS 2378 primer, whereas the lowest number (7) was scored for PBS 2376. The average number of bands per primer was 11.85. Out of all identified loci, across all genotypes, 17 (20.5%) were polymorphic (Table 1, File S1). No species-specific bands of possible diagnostic application were scored. Only in the case of one locus (2253/4) its quasi-diagnostic character was observed, as no amplification product was scored for any tested accessions representing two *Avena* species: *A. atlantica* and *A. longiglumis* (Table S2). The set of PBS primers used in this study generated 13 highly informative loci with PIC value greater than 0.43 (data not shown) which represented 76.47% of polymorphic loci. The average PIC for the particular primer range from 0.04 (PBS 2229) to 0.21 (PBS 2376) with an average value of 0.10.

iPBS markers enabled the detection of polymorphism not only between the tested *Avena* species but also between the majority of accessions representing individual species. The number of iPBS bands ranged from 74 for the *A. insulanis* and *A. sativa*, to 82 for *A. abyssynica*, *A. fatua*, and *A. nuda*. The highest rate of polymorphic bands was scored for accessions representing *A. fatua* (18.07%) and *A. nuda* (16.87%), whereas the lowest number of polymorphic bands was observed for *A. hirtula* and *A. sativa* (1.20%). In order to estimate the genetic diversity of studied *Avena* species, Shannon’s information index (I) and expected heterozygosity (H_e_) were calculated. Both parameters revealed a low level of gene diversity, with the highest values observed for *A. nuda* (*I* = 0.099; *He* = 0.068), *A. abyssinica* (*I* = 0.091; *He* = 0.063), and *A. fatua* (*I* = 0.093; *He* = 0.062). No polymorphism was scored among accessions representing *A. atlantica*, *A. longiglumis*, *A. insulanis*, and *A. sterilis* (Table 2)*.*

**Table 2:** Genetic variation revealed by iPBS markers of 60 accessions representing 13 *Avena* species

Section	Species	N_A_	2n	Genome	%P	I	He
*Agraria*	*A. strigosa*	6	14	As	2.41%	0.016	0.012
	*A. nuda*	4	14	As	16.87%	0.099	0.068
*Tenuicarpa*	*A. damascena*	5	14	Ad	2.41%	0.012	0.008
	*A. atlantica*	2	14	As	0.00%	0.000	0.000
	*A. hirtula*	3	14	As	1.20%	0.006	0.004
	*A. longiglumis*	1	14	Al.	0.00%	0.000	0.000
	*A. barbata*	4	28	AB	12.05%	0.070	0.048
*Ethiopica*	*A. abyssinica*	6	28	AB	14.46%	0.091	0.063
*Pachycarpa*	*A. insulanis*	2	28	AC	0.00%	0.000	0.000
*Avena*	*A. sterilis*	7	42	ACD	0.00%	0.000	0.000
	*A. fatua*	5	42	ACD	18.07%	0.093	0.062
	*A. sativa*	6	42	ACD	1.20%	0.008	0.006
*	*A. byzantiana*	9	42	ACD	4.82%	0.019	0.012

*N*_*A*_, number of accessions; *P*, percentage of polymorphic loci; *I*, Shannon’s Information Index; *He*, expected heterozygosity; * *A. byzantina* was not included in taxonomy of genus *Avena* described by Baum (Baum 1977)

**Fig. 1:**
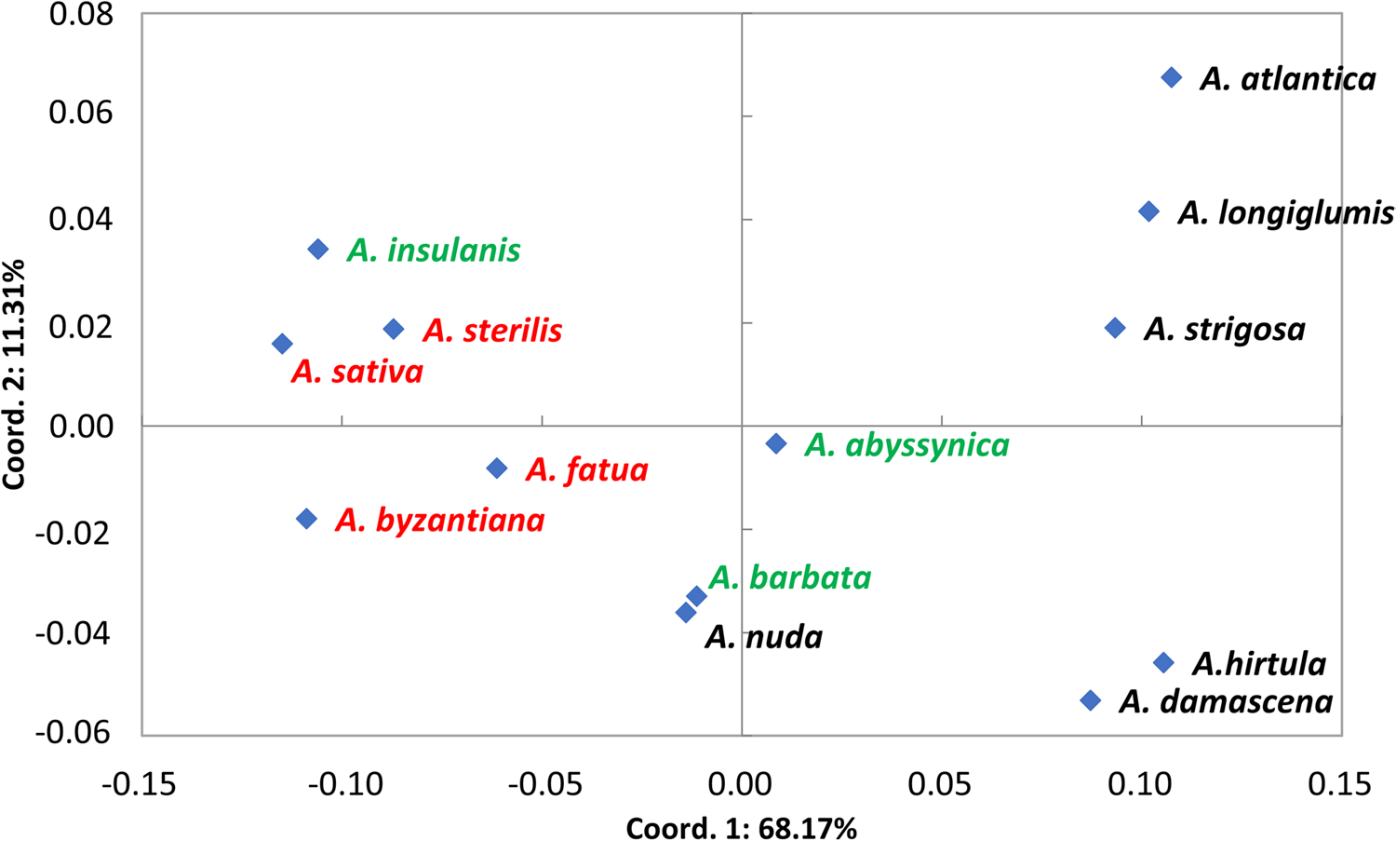
Projection of studied *Avena* species on the first two axes after principal coordinates analysis (PCoA) based on Nei’s genetic distance values

**Fig. 2:**
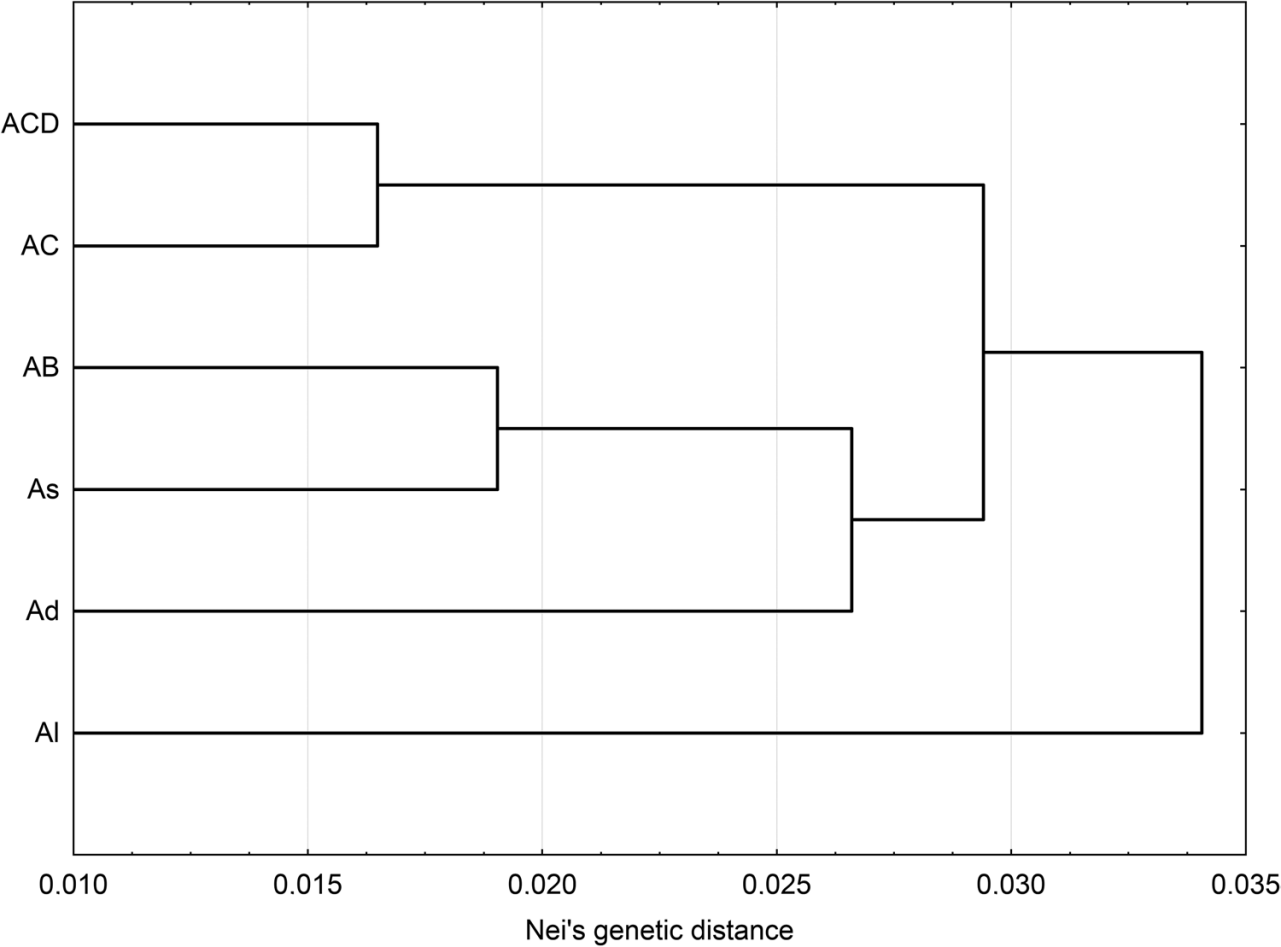
Genetic association of six genome types of *Avena* based on iPBS data. Genome types and their assignation to particular *Avena* species according to the data located in Table 2

Values of Dice similarity coefficient estimated between all pairs of accessions (Table S3) suggested low genetic differentiation within studied material, as the minimum value of that coefficient was 0.893, which described the relation between *A atlantica* and seven out of nine A*. byzantine* accessions and between *A hirtula* 51847 (IHAR) and *A. sativa* 52267 (IHAR). The UPGMA clustering of accessions based on the Dice similarity coefficient was performed and based on the results a cluster tree was plotted (Fig. S1). The analyzed *Avena* accessions were clustered generally according to their species name. Furthermore, two clusters could be distinguished in the presented tree. Cluster I consisted of 25 accessions representing the following *Avena* species: *A. strigosa* (6), *A. longiglumis* (1), *A. atlantica* (2), *A. hirtula* (3), *A. damascena* (5), *A. nuda* (2), *A. abyssynica* (3), *A. barbata* (2), and *A. fatua* (1). Cluster II gathered 35 accessions representing *A. abyssynica* (3), *A. nuda* (2), *A. barbata* (2), *A. sterilis* (7), *A. fatua* (4), *A. insulanis* (2), *A. byzantiana* (9), and *A. sativa* (6)*.* Based on the polymorphism revealed by iPBS markers, a matrix of Nei’s genetic distances (D_N_) between analyzed *Avena* species was also created and subjected to Principal coordinate analysis (PCoA). The Nei’s genetic distance values ranged from 0.010 (*A. sativa* vs*. A. byzantiana*) to 0.199 (*A. atlantica* vs*. A. byzantiana*). The results of PCoA revealed that 85.88% of the genetic variability of the analyzed species was explained by its first three components (68.17%, 11.31%, and 6.4%, respectively). Figure 1 illustrates the dispersion of the analyzed *Avena* species on the plane defined by the first two axes. The results of PCoA distinguished three groups of species clearly dispersed along the Coord.1. The first group included *A. atlantica*, *A. longiglumis*, *A. strigosa*, *A. hirtula*, and *A. damascena*. In turn, *A. sativa*, *A. sterilis*, *A. fatua*, *A. byzantina*, and *A. insularis* formed the second group. In the central part of the graph, one could find *A. barbata,* *A. nuda,* and *A. abyssynica*. In order to estimate the relationships between *Avena* genome types, individuals were first grouped according to the defined genome type, and then Nei genetic distance (D_N_) values between these groups were estimated. Obtained D_N_ values ranged from 0.019 (As vs. AB genome) up to 0.148 (Al vs. ACD) and 0.170 (Al vs. AC). The results of UPGMA clustering based on D_N_ values, divided the studied *Avena* genome types into three clusters. Al genome appeared as the most distinct, solitary cluster. The second cluster gathered Ad, As, and AB genomes, whereas the third cluster consisted of AC and ACD genomes (Fig. 2).

The results of AMOVA showed that most of the detected variability (66.68%) was located between the analyzed *Avena* species, while the remaining 33.32% determined the diversity between the accessions representing individual species (Table 3). In the case when the sections of *Avena* species proposed by Baum (1977) were considered, 35.56% of the variation was found among sections, 30.29% of the total variation resided among species within sections, whereas the remaining 34.14% was present within individual species. According to the values of average gene diversity over loci, the most diverse section was Ethiopica, followed by Agraria, Tenuicarpa, Avena, and Pachycarpa (Table 4). For the latter, only one *Avena* species (*A. insulanis*) represented that section in our study, and no polymorphism was found among *A. insulanis* accessions, thus π_*n*_ was 0. For the ploidy level of *Avena* species, the highest proportion of the total variation was found among groups differed in terms of ploidy level (47.88%), whereas variation among species within these groups and within species was 23.70% and 28.42%, respectively (Table 3). Estimated π_*n*_ values showed that tetraploid species were the most differentiated and were followed by diploid and hexaploidy species (Table 4). Finally, when the genome types of *Avena* species were considered almost half (49.02%) of the total variation was found among these types, whereas 21.52% was present among species within these groups and 29.45% resided within species (Table 3). The π_*n*_ values estimated for each genome type revealed that the most diverse genome type was AB, followed by As, ACD, Ad, AC, and Al (Table 4). For AC and Al genome types, π_*n*_ values were 0.0 due to the fact that in our study, only one *Avena* species (*A. longiglumis*) represented by one accession represented the Al genome type, whereas in the case of AC genome type, only one *Avena* species (*A. insulanis*) represented that genome type, and no polymorphism was found among accessions representing that species.

**Table 3:** Partitioning of diversity found in studied *Avena* accessions using AMOVA

	Source of variation	df	Sum of squares	Variance components	Percentage of variation	p
Species^a^	Among species	11	152.194	2.589	66.68	< 0.0001
	Within species	47	60.789	1.293	33.32	–
Section^b^	Among groups	4	84.512	1.493	35.56	< 0.05
	Among species within groups	7	45.667	1.272	30.29	< 0.0001
	Within species	39	55.900	1.433	34.14	–
Ploidy^c^	Among groups	2	96.862	2.179	47.88	< 0.001
	Among species within groups	10	60.316	1.079	23.70	< 0.0001
	Within species	47	60.789	1.293	28.42	–
Genome type^d^	Among groups	5	114.674	2.15281	49.02	< 0.0001
	Among species within groups	7	42.504	0.94511	21.52	< 0.0001
	Within species	47	60.789	1.29338	29.45	–

^*a*^*F*_*ST*_ = 0.6668; *A. longiglumis* represented in our study by only one accession was excluded from the analysis

^*b*^*F*_*ST*_ = 0.6585; *A. byzantina* which was not included in *Avena* systematics proposed by Baum (1977) was excluded from the analysis

^c^*F*_*ST*_ = 0.7158

^d^*F*_*ST*_ = 0.7055

**Table 4:** Group-specific values of average gene diversity over loci (π_*n*_)

Section	π_*n*_	Ploidy	π_*n*_	Genome	π_*n*_
Agraria	0.069	Diploid	0.058	Al	0.0^a^
Avena	0.035	Tetraploid	0.078	Ad	0.012
Ethiopica	0.076	Hexaploid	0.033	As	0.062
Pachycarpa	0.0^b^			AB	0.078
Tenuicarpa	0.061			AC	0.0^b^
				ACD	0.033

^a^Only one *Avena* species (*A. longiglumis*) represented by one accession represented that genome type

^b^Only one *Avena* species (*A. insulanis*) represented that section (Pachycarpa) and genome type (AC) in our study; no polymorphism was found within that species

### Soluble carbohydrates

**Fig. 3:**
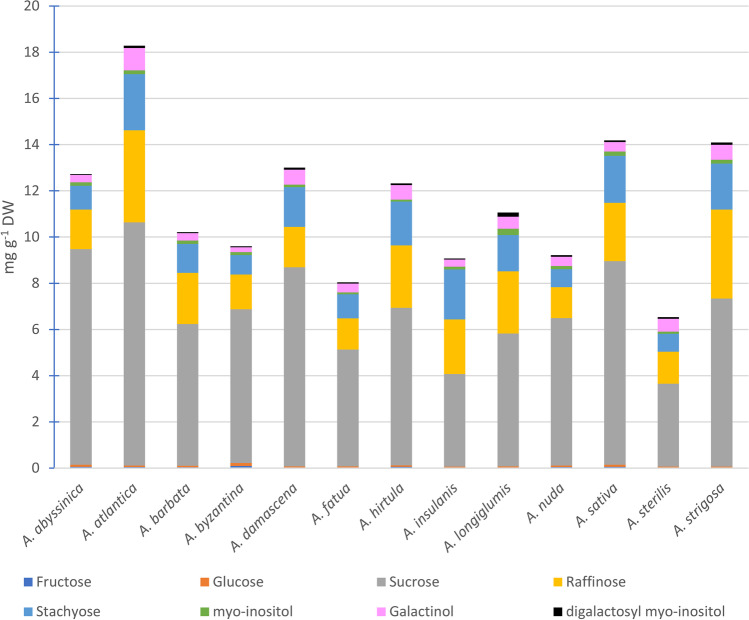
Composition of soluble carbohydrates fraction in caryopses of studied *Avena* species

In caryopses of all studied *Avena* species, the following soluble carbohydrates were identified: sucrose, raffinose family oligosaccharides (RFOs, represented by raffinose and stachyose), *myo*-inositol, and its a-d-galactosides (galactinol and digalactosyl *myo*-inositol), fructose and glucose. Despite the identical qualitative composition, the analyzed oat species revealed high variation in the content of soluble carbohydrates, both among accessions representing each species (Table S4, Fig. S2) as well as between species (Fig. 3, Table 5). The content of total soluble carbohydrates ranged from 6.54 ± 1.43 to 18.28 ± 1.00 mg g^−1^ of dry weight (DW), in *A. sterilis* and *A. atlantica*, respectively. Sucrose, the major sugar (Fig. 3), accounted for more than 50% of total carbohydrates in most oats species/accessions. The highest content of sucrose (ca 8–10 mg g^−1^ DW) was found in caryopses of *A. atlantica*, *A. abyssinica*, and *A. sativa*, whereas the lowest (ca 3–4 mg g^−1^ DW) in *A. sterilis* and *A. insulanis* (Fig. 3). The next to sucrose was raffinose and stachyose, occurring together at a level as high as sucrose (in *A. insulanis*), slightly lower (in *A. strigosa*, *A. hirtula*, and *A. longiglumis*), or even 3–4-fold lower (in *A. abyssinica*, *A. byzantina*, *A. fatua*, and *A. nud*a, Fig. 3). Regardless of the species, raffinose dominated above stachyose (except for 6 among 47 accessions; Table S4). *Myo*-inositol and its galactosides were less abundant carbohydrates (0.28–1.44 mg g^−1^ DW), sharing only a few % of total soluble carbohydrates in most species (Table S4). Monosaccharides accounted for no more than 1% of all soluble carbohydrates, with except for *A. abyssinica* (1.12%), *A. nuda* (1.19%), and *A. byzantina* (2.3%).

**Table 5:** The content of soluble carbohydrates in caryopses of studied *Avena* species.

Carbohydrates	Species												
	*A. abyssinica*	*A. atlantica*	*A. barbata*	*A. byzantina*	*A. damascena*	*A. fatua*	*A. hirtula*	*A. insularis*	*A. longiglumis*	*A. nuda*	*A. sativa*	*A. sterilis*	*A. strigosa*
	Monosaccharides and sucrose												
Fructose	0.056^ab^	0.050^ab^	0.041^ab^	0.093^b^	0.023^a^	0.029^ab^	0.056^ab^	0.026^ab^	0.029^ab^	0.045^ab^	0.059^ab^	0.025^a^	0.023^a^
Glucose	0.086^ab^	0.058^ab^	0.057^ab^	0.128^b^	0.047 ^ab^	0.044^a^	0.063 ^ab^	0.039^a^	0.046^ab^	0.064 ^ab^	0.084^ab^	0.039^a^	0.046^a^
Sucrose	9.333^d^	10.524^d^	6.140^abcd^	6.655^abcd^	8.615^bcd^	5.058^abc^	6.819^abcd^	4.002^ab^	5.750^abcd^	6.379^abcd^	8.810^cd^	3.591^a^	7.272^bcd^
	Raffinose family oligosaccharides (RFOs)												
Raffinose	1.713^a^	3.988^c^	2.212^ab^	1.502^a^	1.756^ab^	1.349^a^	2.704^b^	2.364^ab^	2.693^b^	1.345^a^	2.527^b^	1.382^a^	3.852^c^
Stachyose	1.037^ab^	2.431^e^	1.256^abcd^	0.841^a^	1.713^abcde^	1.043^abc^	1.895^bcde^	2.166^de^	1.569^abcde^	0.773^a^	2.038^de^	0.778^a^	1.985^cde^
	*myo*-inositol and its galactosides												
*Myo*-inositol	0.143^bcd^	0.163^cd^	0.146^bcd^	0.127^abc^	0.113^abc^	0.078^a^	0.084^a^	0.117^abc^	0.276^e^	0.135^abcd^	0.182^d^	0.094^ab^	0.171^cd^
Galactinol	0.315^a^	0.964^c^	0.313^a^	0.214^a^	0.647^bc^	0.383^ab^	0.631^bc^	0.310^a^	0.521^ab^	0.406^ab^	0.410^ab^	0.553^b^	0.642^bc^
Di-galactosyl-*myo*-inositol	0.043^a^	0.102^bcd^	0.051^ab^	0.043^a^	0.091^abcd^	0.057^abc^	0.069^abc^	0.052^abc^	0.177^d^	0.073^abc^	0.076^abc^	0.075^abc^	0.105^cd^
Sum of soluble sugars	12.726^cd^	18.282^e^	10.217^bc^	9.602^b^	13.005^cd^	8.041^ab^	12.320^cd^	9.077^ab^	11.061^bcd^	9.220^b^	14.186^d^	6.537^a^	14.096^d^
Sum of monosaccharides	0.143^b^	0.109^ab^	0.099^ab^	0.221^c^	0.070^ab^	0.073^ab^	0.119^ab^	0.065^ab^	0.075^ab^	0.109^ab^	0.143^b^	0.064^a^	0.068^ab^
Sum of RFOs	2.750^ab^	6.419e	3.468^bc^	2.343^a^	3.469^bcd^	2.392^a^	4.599^d^	4.530^cd^	4.262^cd^	2.118^a^	4.565^d^	2.160^a^	5.837^e^
Sum of *myo*-inositol and its galactosides	0.500^ab^	1.230^f^	0.510^ab^	0.383^a^	0.851^de^	0.518^ab^	0.784^cde^	0.479^ab^	0.974^ef^	0.614^bc^	0.667^bcd^	0.722^cd^	0.918^e^

Values are means (mg per g dry matter). Data were processed by analysis of variance, values with different superscripts (a–e) differ significantly (*p* < 0.05) in Tukey’s test (comparison valid within rows only). *A. longiglumis* SD was represented in our study by only one accession

**Fig. 4:**
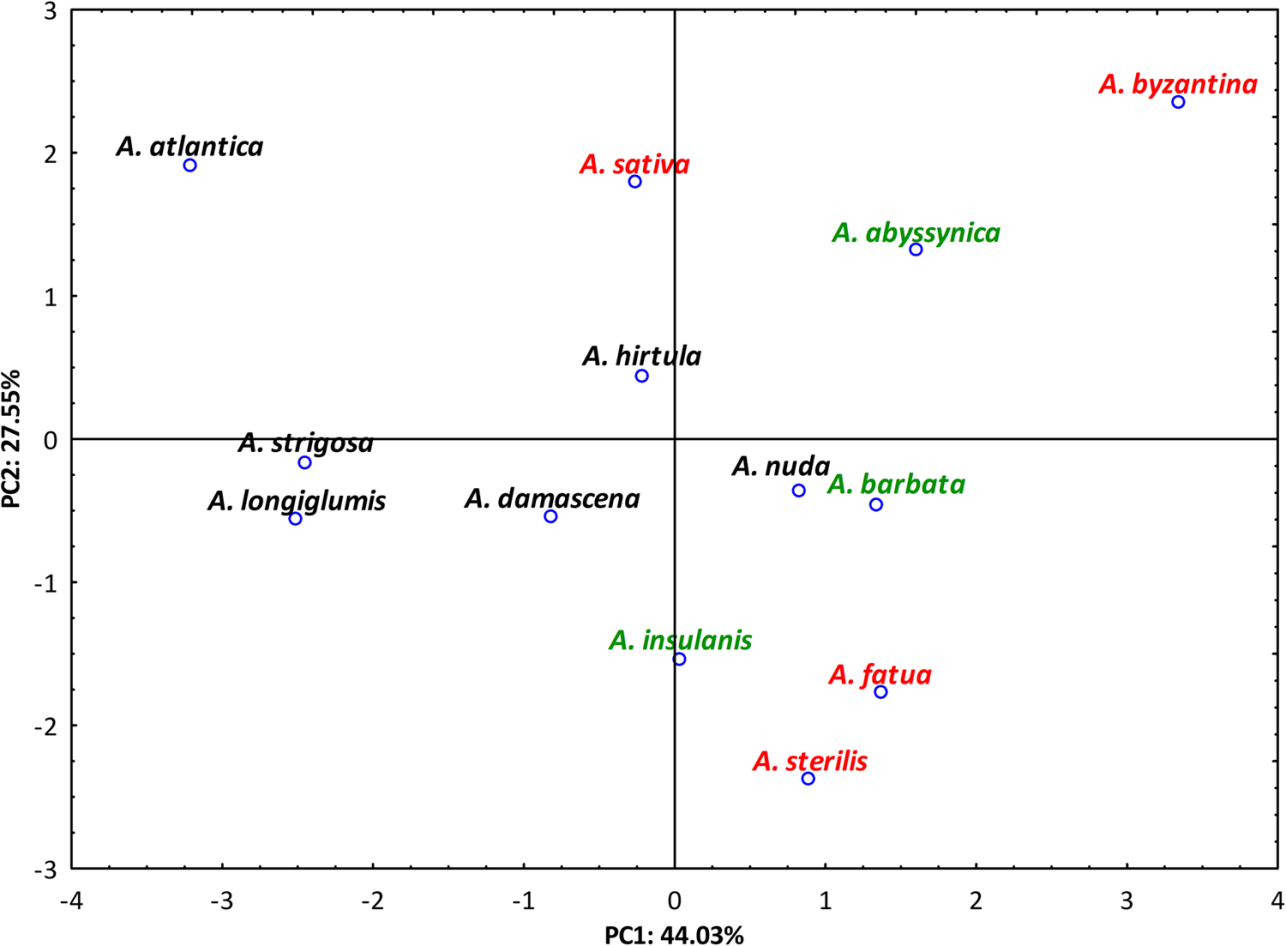
2D PCA-plot showing clustering of 13 *Avena* species based on data for soluble carbohydrates. Diploid *Avena* species are marked with black color, tetraploid species are marked with green, whereas hexaploid species are marked with red color

The results of the principal components analysis (PCA) indicated that 86.75% of the variability in the content of soluble carbohydrates in the analyzed species is explained by its first three components (44.03%, 27.55%, and 15.17%, respectively). The content of RFOs (raffinose and stachyose) as well as galactinol and di-galactosyl *myo*-inositol had the greatest influence on the dispersion of the analyzed samples against PC1. In the case of PC2, these were glucose, fructose, and sucrose (given in descending order), whereas in the case of PC3 *myo*-inositol had a decisive role in discriminating of the analyzed oat accessions. The projection of analyzed *Avena* species on the first two axes was shown in Fig. 4. The diploid *Avena* species (black) were scattered mainly on the left from PC1 and in the central part of Fig. 4 (with *A. atlantica* diverged along the PC1). The hexaploid *Aven*a species marked with red color (*A. sativa*, *A. byzantina*, *A. fatua*, and *A. sterilis*) could be found in the peripheral areas of the plane defined by PC1 and PC2 axes, with *A. byzantina* that diverged along PC1. The tetraploid *Avena* species (*A. abyssynica*, *A. barbata*, and *A. insulanis*, marked with green color) occupied the area between the two mentioned above groups of species.

## Discussion

For centuries, oat, which served mainly as forage and less frequently as food, was treated as a minor cereal which was cultivated predominantly on poor soils unsuitable for the cultivation of other cereals (Moore-Colyer 1995). Currently, the oat is the sixth most cultivated cereal in the world; however, recently, there has been observed an increase in interest in oat as a trendy, healthy functional food (Hofmann 1995; Andersson and Börjesdotter 2011). The growing interest in oat revealed the need for intensification of breeding programs which aimed at providing new varieties characterized by higher yield, improved biochemical composition of grain, increased resistance to pests and/or abiotic stresses, etc.

Comprehensive characteristics of breeding material are a crucial element for the planning of effective and efficient breeding programs as it allows precise evaluation of genetic variation available to breeders (Govindaraj et al. 2015). Although the evaluation of genetic variation in oat is less intensively studied in comparison with other cereal crops, there are a number of papers describing genetic diversity and differentiation of various *Avena* species and accessions. Initially, the polymorphism of several isoenzyme systems was used for the genetic characteristic of *Avena* (Marshall and Allard 1970; Craig et al. 1972; de la Hoz and Fominaya 1989). Later on, different DNA marker systems were used for the investigation of polymorphism in oats: restriction fragment length polymorphism (RFLP) (Beer et al. 1993; Nocelli et al. 1999), random amplified polymorphic DNA (RAPD) (Heun et al. 1994; Drossou et al. 2004), amplified fragment length polymorphism (AFLP) (Fu and Williams 2008; Constandinou et al. 2018), inter simple sequence repeats (ISSR) (Boczkowska and Tarczyk 2013; Sharma et al. 2016), or microsatellite markers (SSR) (Li et al. 2000a; Nikoloudakis et al. 2016). Application of these techniques allowed also genetic mapping (O’Donoughue et al. 1992; Portyanko et al. 2001), resolving the questions of phylogeny and systematic position of species (Alicchio et al. 1995; Li et al. 2000b), and identification of molecular markers associated with certain traits, which were then used for fast and efficient selection of breeding material (Rines et al. 2006). One of the available genetic marker system which is increasingly used in plant genetics and breeding is iPBS (Kalendar et al. 2010). So far, this molecular technique was successfully applied in genetic studies of, e.g., guava (Mehmood et al. 2016), potato (Demirel et al. 2018), barley (Bonchev et al. 2019), oregano (Karagoz et al. 2020), peach, and nectarine (Naeem et al. 2021). TEs, due to their mobile nature, had a significant impact on the organization, plasticity, and evolution of genomes (Frost et al. 2005). Therefore, the retrotransposon-based iPBS markers appeared as an ideal candidate molecular tool suitable for genetic diversity and evolutionary studies of genus *Avena*, which although extensively studied still remain elusive (Fu 2018).

### Genetic diversity revealed by iPBS markers

In the current paper, iPBS markers proved their usefulness in genetic studies of the genus *Avena*. Application of that retrotransposon-based molecular technique not only allowed to trace genetic variation among studied *Avena* accessions but also provided valuable data which may become useful in the comprehensive study of the evolution of the whole genus. iPBS genotyping of our *Avena* collection revealed that 20.5% of amplified fragments (bands) were polymorphic. The highest polymorphism was found among accessions representing *A. fatua* (18.07%) and *A. nuda* (16.87%), whereas no variation was observed between genotypes representing *A. atlantica*, *A. longiglumis*, *A. insulanis*, and *A. sterilis*. Although in our study, individual *Avena* species were represented by an unequal number of accessions, the level of polymorphism revealed for particular species seems not to be associated with the sample size: both species represented by the smallest number of accessions (*A. longiglumis*, *A. atlantica*, *A. insulanis*) as well as these represented by the highest number of genotypes (*A. byzantiana* and *A. sterilis*) can be found among species with the lowest polymorphism. Fu and Williams (2008) in their AFLP studies of 25 *Avena* species reported 7.3% to 62.7% of polymorphic bands for a species. Moreover, the authors noticed a positive correlation between the number of polymorphic bands and the number of accessions representing particular species.

So far, iPBS markers were used only once in genetic studies of *Avena* species (Nečajeva et al. 2021). The study revealed that the proportion of polymorphic bands within eighteen Latvian, one Polish, and one Norway *A. fatua* populations ranged from 47 to 85%, but when the number of polymorphic fragments revealed by a particular PBS primer was considered, the values were even higher and ranged from 88.9 to 100%. Application of other molecular tools in previously published genetic studies of various *Avena* species revealed the differentiated level of polymorphism. For example, analyses of 16 *Avena* species with the use of RAPD markers yielded from 62.5 to 100% polymorphic bands per primer (Sood et al. 2016), whereas even 90% to 100% of RAPD polymorphic bands per primer was reported by Drossou et al. (2004) during the study of relationships between 18 *Avena* species. Application of AFLP markers allowed to identify of about 42.8% of polymorphic bands during the analysis of the genetic diversity of 96 oat cultivars (Fu et al. 2003), but there are also examples in which AFLP markers can reveal up to 97.4% (Drossou et al. 2004) of polymorphic loci in analyzed *Avena* species. In the case of ISSR markers, the revealed polymorphism varies between 41.7% (Paczos-Grzęda 2007) and 70.9% (Paczos-Grzęda et al. 2009) in the studies of *A. sativa* and *A. sterilis*, respectively, whereas application of codominant SSR markers allowed to observe even 100% of polymorphic loci (Sood et al. 2016). Although the reported in this paper’s level of polymorphism of studied *Avena* collection was lower than in the case of other mentioned molecular tools, it appeared to be sufficient to distinguish analyzed *Avena* species and the majority of accessions. Our observations, together with the results of the study by Nečajeva et al. (2021) proved that iPBS markers should be treated as a cost-efficient and reliable source of molecular data in genetic studies of *Avena* species and noteworthy alternative for RAPD markers, which are said to suffer from a low reproducibility of the results (Kumari and Thakur 2014), laborious and technically demanding AFLP markers (Karaca and Ince 2017), or SSRs which are time-consuming and expensive to develop (Yang et al. 2013).

The current study showed that most of the iPBS variation was located among studied *Avena* species, among groups of *Avena* species which belong to different genome types, among groups of *Avena* species which share the same ploidy level, and among *Avena* sections proposed by Baum (1977). The groups above are given in descending order of the value of the percentage of variation identified among them. Only about one-third of the total genetic variation resided within individual species. These observations are congruent with the results described by Fu and Williams (2008) who also reported that most of the total genetic variation is partitioned among studied *Avena* species (59.5%) and only about 40.5% within individual species.

UPGMA clustering of 60 *Avena* accessions based on the Dice’s similarity coefficient generated a dendrogram in which two main clusters were clearly distinguished. Cluster I, which gathered 25 accessions, included genotypes representing all diploid *Avena* species, two tetraploid oats (*A. abyssynica* and *A. barbata*), and one accession representing hexaploid *A. fatua*. The remaining 35 *Avena* accessions could be found within cluster II, which gathered genotypes representing all hexaploid *Avena* species together with tetraploid *A. abyssynica* and *A. insulanis* and two accessions of diploid *A. fatua* and one accession of tetraploid *A. barbata*. High genetic similarity between *A. sativa*, *A. byzantiana*, *A. sterilis*, and *A. fatua* revealed here by iPBS markers is concordant which previous observations concerning these hexaploid *Avena* species. As mentioned above, four oat species have identical ACD genome type, and share similar morphology and similar C-banding pattern of chromosomes (Rajharthy and Thomas 1974; Badaeva et al. 2011). Available results of molecular studies (Li et al. 2000b; Fu and Williams 2008) as well as observations of free crosses between these species (Loskutov 2001) also confirm their close evolutionary relationships. High similarity between tetraploid *A. insulanis* (with AC genome) and four hexaploid oat species with the ACD could be also noticed. This observation is in agreement with the previous findings by Rajharthy and Thomas (1974), who stated that tetraploid AC-genome species were sources of the hexaploid oat A and C genomes. Moreover, the lack of significant differences in the banding pattern of chromosomes between *A. insularis* and two other tetraploid oats (*A. magna* and *A. murphyi*) confirmed the major role of AC genome teraploids in the evolution of hexaploid *Avena* species (Jellen and Ladizinsky 2000). When two other studied tetraploid oats *A. abyssynica* and *A. barbata* representing the AB genome group were considered, their higher genetic differentiation was observed (based on the pattern of the scattering of their accessions between cluster I and cluster II), as well as their closer relationship with the diploid oats. The close relationship between AB and A genome *Avena* species was reported also by Fu (2018) based on an analysis of chloroplast SNPs. Phylogenetic studies suggest that the most plausible ancestor of the A genome for *A. abyssynica* and *A. barbata* is an As-genome species; however, the second ancestor of these two species has not been identified yet, but most likely, it does not belong to the As-genome group (Loskutov and Rines 2011). According to the available literature, it could be *A. damascena* as it was proposed for *A. agadiriana*, another AB genome tetraploid (Badaeva et al. 2010). This theory may be supported by our findings where *A. damasecna* can be found in one subcluster within cluster I together with *A. abyssynica* and *A. barbata*. Furthermore, all diploid *Avena* species were gathered in one cluster, confirming their common genetic features reported previously by the means of molecular (Fu and Williams 2008) and cytological studies (Rajharthy and Thomas 1974; Yan et al. 2016). Finally, although the accessions representing a particular species or shearing the same type of genome tend to cluster together, there were some samples representing, e.g., A*. nuda* and *A. fatua*, which did not follow that rule. Scattering of these accessions between different *Avena* clusters may be interpreted as their higher genetic variation revealed by iPBS markers and/or not clear relationships of the particular taxon with other oats as it is in the case of *A. nuda* (Baum 1977). The analogical situation, i.e., clustering of some genotypes representing particular *Avena* species separately, with other oats, was also observed, e.g., by Li et al. (2009) by the means of consensus chloroplast SSRs and Sood et al. (2016) based on the results of RAPD and SSR markers.

Additionally, to infer about relationships between studied *Avena* species, the PCoA was applied based on Nei’s genetic distance values estimated between all pairs of oat species. Also in this approach, the observed pattern of genetic variation clearly corresponded to the ploidy level of the analyzed species, pointing on a different character of diploid and hexaploid *Avena* species which were grouped separately, and intermediate character of tetraploid oats, in which AB genome oats (*A. abyssynica* and *A. barbata*) were more similar to diploid oats, but *A. insularis* (AC genome) revealed close affinity to hexaploid *Avena* species. As in both mentioned above approaches, i.e., when individual *Avena* accessions were considered or the species level was analyzed, the genome type and ploidy level appeared to play the major role in the clustering process, the additional analysis was performed in which the accessions were grouped according to their genome type. Also, in this analysis, the AC genome tetraploids revealed the closest relationships with ACD genome hexaploids, whereas AB genome tetraploids were clustered with As and Ad diploids. The Al genome diploid appeared here as the most distinct. Our results confirmed previous observations of Katsiotis et al. (1997) who reported that the A and B genomes reveal close relationships and that the *barbata* group of teraploids (AB genome) arose from As diploids through autoteraploidization. The high affinity between As genome and AB genome tetraploid was reported also by Nikoloudakis et al. (2008). According to the authors (Nikoloudakis et al. 2008), the distinction between the A and B genomes was not possible, while the major genetic divergence between the A and C genomes was observed. C genome appeared to be approximately 15% longer than the A genome (Yan et al. 2016) most likely due to differences in repetitive DNA content (Jellen et al. 1993). Moreover, mostly asymmetrical, heterochromatic chromosomes are characteristic for *Avena* species carrying the C genome, while symmetrical karyotype and low content of heterochromatin are found in species with A genome (Fominaya et al. 1988).

The revealed pattern of interspecific variation of studied *Avena* species appeared to be concordant also with the grouping of oat species in terms of their crossability proposed by Harlan and de Wet (1971). That gene pool classification system divided the *Avena* species into three groups (gene pools). The primary gene pool, following the Ladizinsky and Zohary (1971), included all cultivated and wild hexaploid oat taxa that cross with each other easily. The secondary gene pool, according to Leggett and Thomas (1995), gathers AC tetraploid species *A. magna*, *A*, *murphhyi*, and *A. insularis* for which successful hybridization with hexaploid *Aven*a species was reported, although some problems with the fertility of the hybrids could be observed. Finally, the tertiary gene pool was distinguished (Leggett and Thomas 1995), which consisted of all diploid *Avena* species and the tetraploid *A. abysinnica*, *A. barbata*, *A. vaviloviana*, and *A. macrostachya*. Although mentioned above, species do not readily hybridize with *A. sativa*, but with the application of some additional in vitro techniques (e.g., embryo rescue), successful trait introgression could be observed (Loskutov and Rines 2011).

### Soluble carbohydrates content and composition

Cereals are cultivated generally for their seeds suitable for food and/or livestock feed. Cereal grains are rich in carbohydrates, with comparatively low content of protein, fats, vitamins, and minerals. In the case of the oat grains, 75–80% of their dry matter are carbohydrates (mostly polysaccharides), whereas protein content is about 11–15% (Rodehutscord et al. 2016), but there are also observations according to which protein content in wild oat species may reach 27–28% (Campbell and Frey 1972) or even 35% (Frey 1975). In addition to proteins, oat grains can be rich also in fats, as it was observed in some diploid and tetraploid species, for which the content of oil in grains may reach up to 12–13% (Welch and Leggett 1997). When the carbohydrate content in oat grains is considered, the main focus of scientists is generally focused so far on the starch and β-glucans because of their role in grain digestibility or beneficial effects on human health (e.g., Davidson et al. 1991). The starch comprises the largest fraction of the carbohydrates and shares up to 73.4% of grains’ dry weight, as it was observed in *A. sativa* (Beloshapka et al. 2016), whereas β-glucans content may vary from 2.2 to 11.3%, depending on the species (Welch et al. 2000). In case of soluble carbohydrates, their qualitative and quantitative composition in oat grains was less frequently studied.

The results of our study showed the usefulness of intra- and inter-specific variation in soluble carbohydrate profiles in caryopses for the characterization of the *Avena* species. The results of capillary gas chromatography showed that all studied *Avena* accessions had the same qualitative composition of soluble carbohydrate profiles, but with significant quantitative differences between individual components. Sucrose appeared to have the largest share in the total carbohydrate content, regardless of the analyzed accession or species. According to our data, sucrose constitutes on average 59.9% of all identified sugars reaching the highest values in *A. atlantica* (73.3%), *A. byzantina*, and *A. nuda* (69.3% and 69.2%, respectively). The highest content of sucrose among free sugars and oligosaccharides in seeds of 22 grass species (MacLeod and McCorquodale 1958) as well as different cereal grains (MacLeod and Preece 1953) was also reported. Oat with 639 mg of sucrose per 100 g of grain took the last position in this ranking after rye, barley, wheat, and maize (1857 mg, 908 mg, 836 mg, and 783 mg, respectively; MacLeod and Preece 1953), but also was found in the middle of the rank (11th out of 22) of the studied grass species for which the sucrose content ranged from 194 mg for *Agropyron repens* up to 10700 mg for *Spartina townsendii* (MacLeod and McCorquodale 1958). Glucose and fructose were also encountered in all analyzed *Avena* accessions but these monosaccharides comprise on average only 0.96% of total identified carbohydrates. Both papers cited above (MacLeod and Preece 1953, MacLeod and McCorquodale 1958) also reported the presence of glucose and fructose in all analyzed seeds, but always as a minor component.

Although plants generally store carbohydrates in long polysaccharide chains (e.g., starch, glucans), sucrose can also be found among storage molecules in roots, leaves, fruits, and seeds. The importance of sucrose is associated also with its central position in plant metabolism, as it is the end product of photosynthesis, the primary sugar transported in phloem and carbon skeleton for the synthesis of organic matter such as amino acids or nucleotides (Stein and Granot 2019). In seeds, sucrose is cleaved into monosaccharides, being the source of carbon skeletons and energy for developing embryo and storage tissues. During seed maturation, sucrose is also a substrate for the synthesis of other types of sucrose-derived oligosaccharides like RFOs (Keller and Pharr 1996) or fructans, present in cereals (Valluru and Van den Ende 2008). In this pathway, *myo*-inositol plays a significant role as the acceptor of galactose moiety in the synthesis of galactinol, a major donor of galactosyl residues for the synthesis of its higher homologues, as stachyose and verbascose (and less abundant ajugose), found in elevated amounts in legumes (Obendorf and Górecki 2012). In cereal grains, raffinose dominates among RFOs, whereas stachyose is present at a very low level (Henry and Saini 1989; Lahuta and Goszczyńska 2010). In our study, raffinose and stachyose were identified in caryopses of all studied *Avena* accessions, with higher raffinose content (raffinose:stachyose ratio ranged from 1.09 for *A. insulanis* up to 1.94 for *A. strigosa*). The total RFOs level was lower only from sucrose and varied from 21.6% (*A. abyssynica*) up to 49.9% (*A. insulanis*) of total carbohydrates. The content of raffinose, stachyose, and sucrose were not correlated; however, the percentage content of sucrose and total RFOs in total soluble carbohydrates revealed a significant negative correlation (*r* = −0.976, *p* < 0.001). The same phenomena was described for barley grains, where the incorporation of sucrose into RFOs reduced the final sucrose pool (Karner et al. 2004).

The central position of galactinol in the synthesis of RFOs was confirmed in our study, as the galactinol content appeared to be positively correlated with the raffinose (*r* = 0.669, *p* < 0.05) and stachyose (*r* = 0.579, *p* < 0.05) level. This is concordant with the observations of Handley et al. (1983), who suggested that the concentration of galactinol is the key factor for the RFOs content. Interestingly, no significant correlation was observed between *myo*-inositol and RFOs contents as it was expected based on the reports in which reduction of *myo*-inositol level decreased galactinol and raffinose in potato (Keller et al. 1998) and soybean (Hitz et al. 2002) or increased level of galactinol was observed in response to elevated *myo*-inositol (Karner et al. 2004). Nevertheless, the reported here low level of *myo*-inositol did not appear as a factor which limited the galactinol and RFOs content in grains of studied *Avena* accessions. The very low level of *myo*-inositol (in most oat accessions below 0.2 mg g^−1^ DW) could be explained by its utilization for the synthesis of phytic acid (Angel et al. 2002).

There are many factors which affect the amount of carbohydrates stored in the seeds, i.e., the availability of substrates for the synthesis of particular carbohydrate and activities of enzymes catalyzing its synthesis and degradation. If we accept that the enzyme balance undergoes genetic control, then the amounts of individual carbohydrates depend on the genetic composition of plants (MacLeod and McCorquodale 1958). As a consequence, the differentiation of carbohydrate profiles can be observed between species, especially when they represent distant lineages, as it was observed by MacLeod and McCorquodale (1958) in their studies of 22 Gramineae species or among legume species (Obendorf and Górecki 2012). In the case of the current study, high quantitative differentiation in carbohydrate profiles was found, both within studied species as well as between them; however, no species-specific carbohydrate fractions were found. But even then, the results of PCA analysis based on obtained carbohydrate data showed an interesting pattern of interspecific variation, in which diploid *Avena* species tend to form a central group, surrounded by the other oats, with hexaploid species scattered generally in the external areas of the 2D PCA-plot, whereas tetraploid species took an intermediate position. However, we are aware of the fact that identified carbohydrates represent no more than 2% of the dry weight of the analyzed grains, so analysis of other components may be also suggested in case of detailed characteristic and/or discriminatory analysis of species representing genus *Avena*.

## Conclusions

Here, we reported the results of the first use of the retrotransposon-based iPBS markers for assessment of the rate of genome divergence within and between species representing genus *Avena*, combined with the analysis of soluble carbohydrate profiles. Both methods applied in our study revealed their discriminatory power for studied *Avena* accessions and allowed to perform their comparison. Molecular data obtained with the use of retrotransposon-based iPBS markers showed that the TEs activity accompanied the *Avena* genome’s natural evolution and domestication. Polymorphism revealed by iPBS markers confirmed previous observations according to which oat species can be differentiated based on their ploidy level, i.e., into a group of diploid and a group of hexaploid species which share the lowest similarity, and a group of tetraploid species with rather intermediate character. iPBS data confirmed also evolutionary relationships between *Avena* genome types revealing high similarity between hexaploid ACD genome and tetraploid AC genome, and close affinity of tetraploid AB genome with diploid A genomes.

Analysis of soluble carbohydrate profiles showed that studied *Avena* accessions share the same quantitative composition, but significant differences in the quantity of particular sugars among studied samples were observed. Application of capillary gas chromatography allowed us to identify a group of ten soluble carbohydrates among which sucrose appeared as the most abundant. Based on the carbohydrate profiles, the relations between studied *Avena* species were studied. The results of PCA analysis revealed an interesting pattern of interspecific variation, convergent to that revealed by iPBS markers. Both molecular and biochemical data presented in our paper not only complement our knowledge about the diversity found within and among *Avena* species, but they may be also used as an important argument in the discussion on the evolution of the whole genus. Such information may also become valuable data useful for breeders who need detailed characteristics of breeding material for the development of successful and efficient breeding programs.

## Supplementary Information

Figure S1. UPGMA clustering of 60 *Avena* accessions based on the values of Dice similarity coefficient (PNG 550 kb)

High Resolution Image (TIF 3187 kb)

Figure S2. The composition of soluble carbohydrates in caryopses of studied *Avena* accessions (PNG 363 kb)

High Resolution Image (TIF 766 kb)

File S1. Raw photographs of agarose gel electrophoresis for all studied *Avena accesions* and all applied PBS primers with description (RAR 17765 kb)

Table S1. List of the *Avena* accessions used in the study. Species arranged alphabetically. (DOCX 18 kb)

Table S2. Binary matrix of iPBS data for 60 *Avena* accessions. (XLSX 26 kb)

Table S3. Dice similarity coefficient values between studied *Avena* accessions (XLSX 24 kb)

Table S4. The content of soluble carbohydrates in caryopses of studied *Avena* accessions (XLSX 22 kb)
