# Supplementary figures and images for: The comparison of polymorphism among Avena species revealed by retrotransposon-based DNA markers and soluble carbohydrates in seeds
Source: J Appl Genet. 2023 Jan 31;64(2):247–64. doi: 10.1007/s13353-023-00748-w (PMC10076396; doi:10.1007/s13353-023-00748-w)

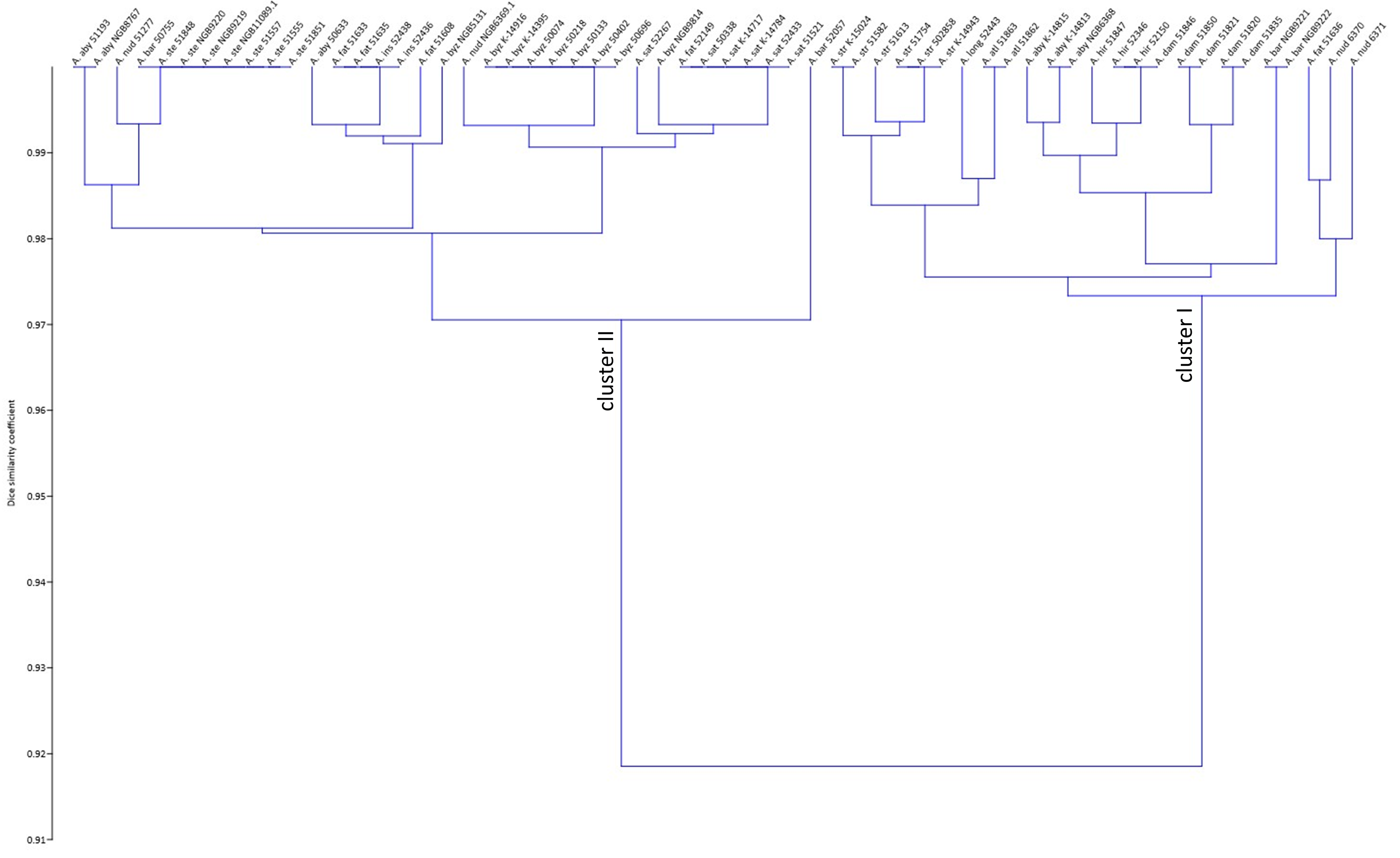

Supplement: Supplementary file 1 — UPGMA clustering of 60 Avena accessions based on the values of Dice similarity coefficient (PNG 550 kb) [file 13353_2023_748_Fig5_ESM.png]

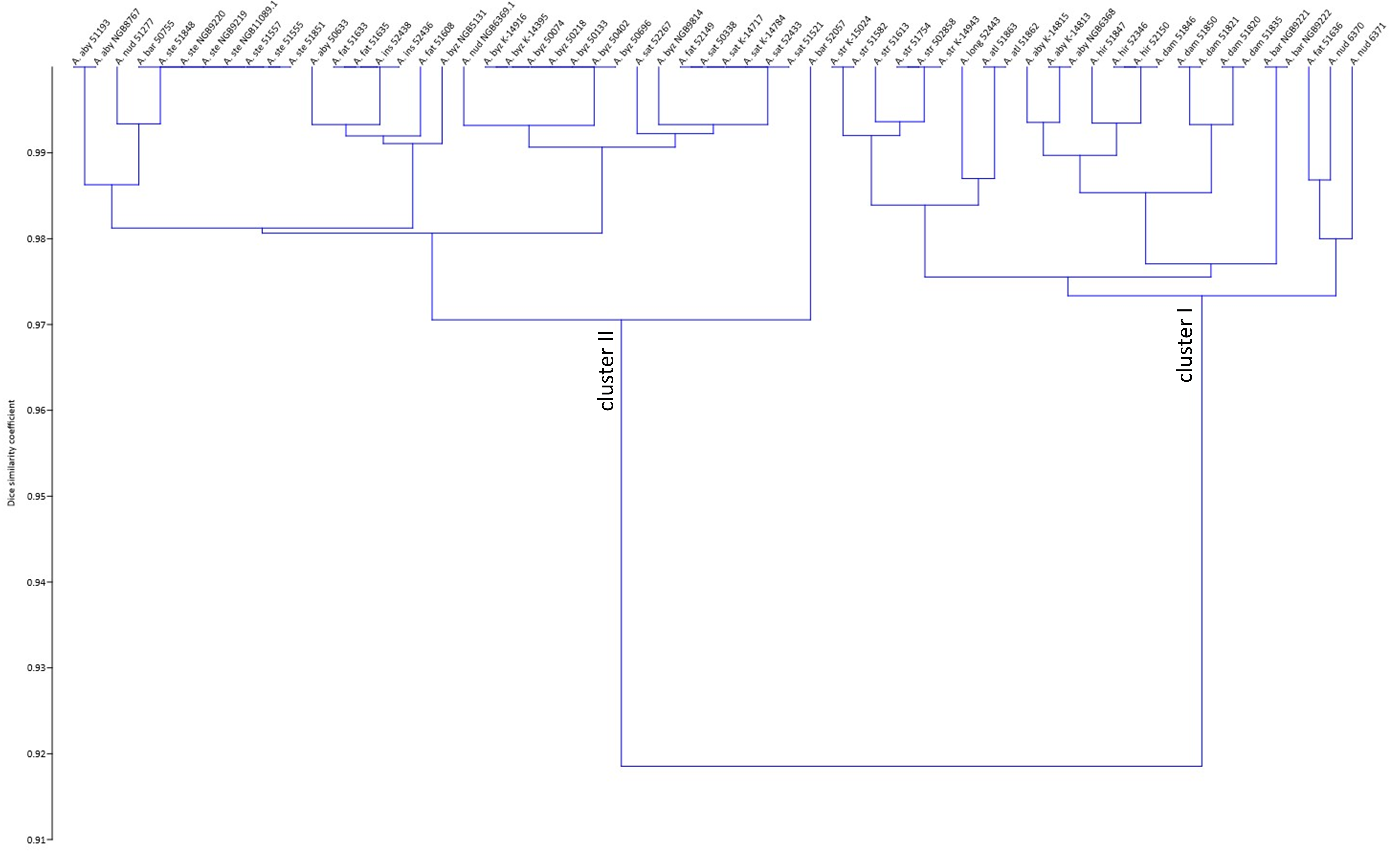

Supplement: Supplementary file 2 — High Resolution Image (TIF 3187 kb) [file 13353_2023_748_MOESM1_ESM.tif]

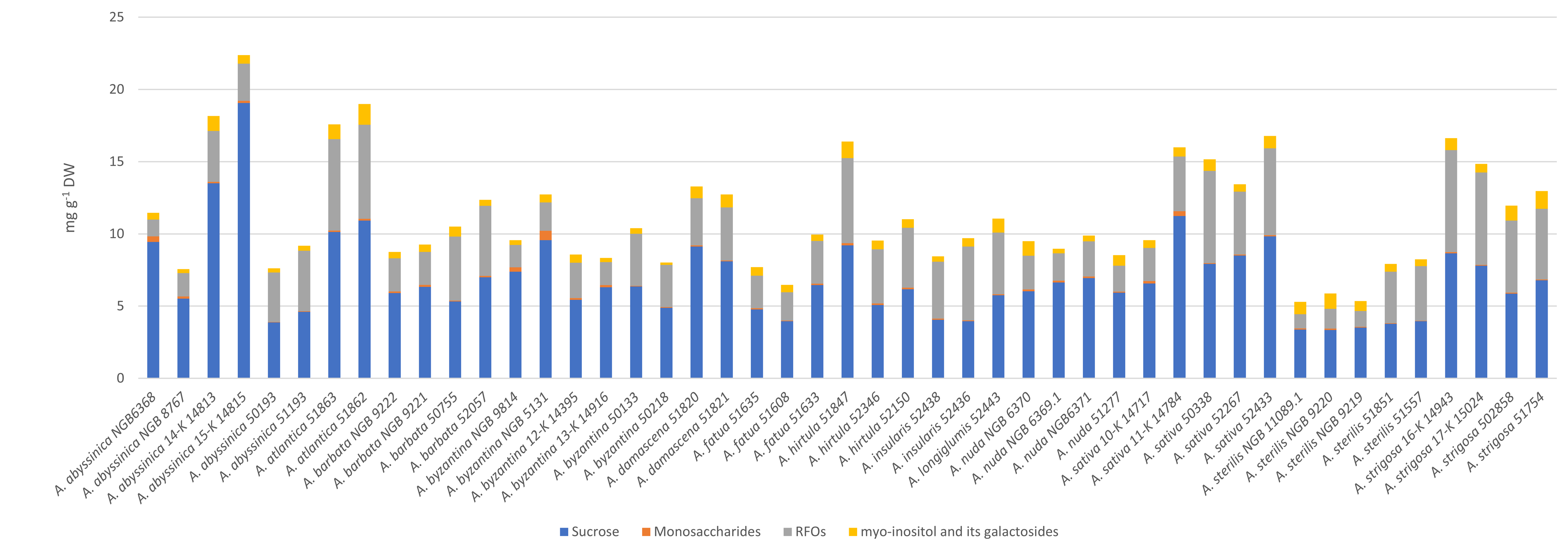

Supplement: Supplementary file 3 — The composition of soluble carbohydrates in caryopses of studied Avena accessions (PNG 363 kb) [file 13353_2023_748_Fig6_ESM.png]

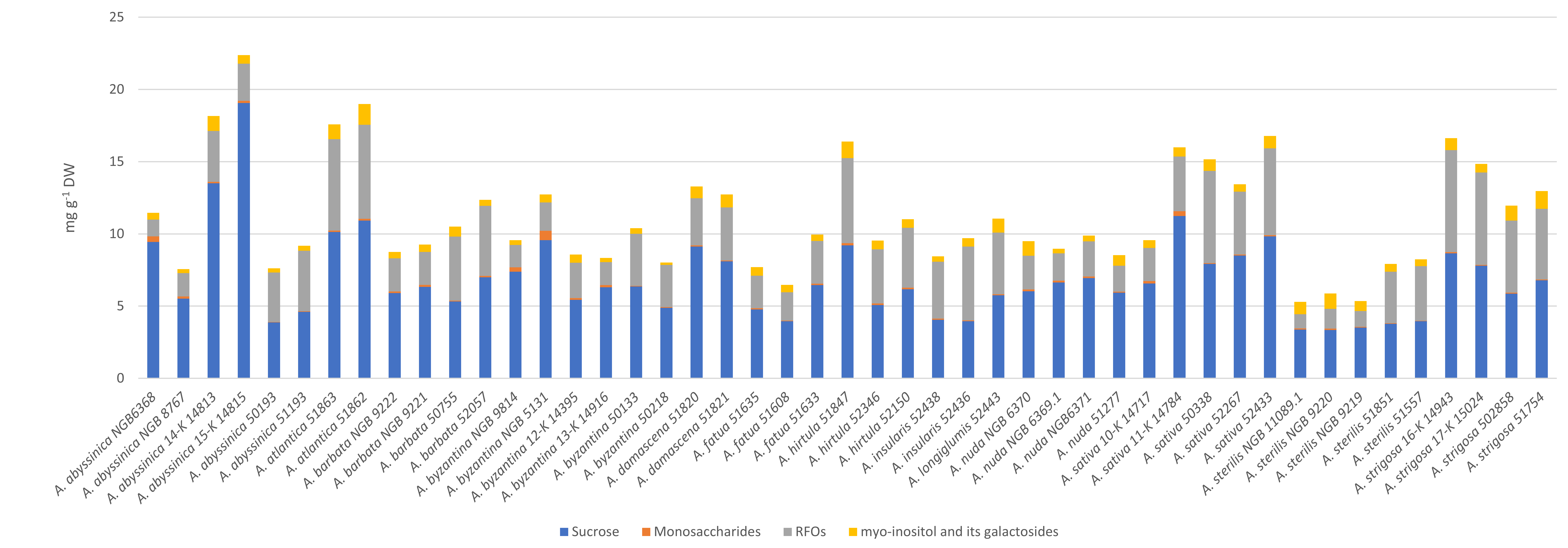

Supplement: Supplementary file 4 — High Resolution Image (TIF 766 kb) [file 13353_2023_748_MOESM2_ESM.tif]
