# Supplementary material for: The comparison of polymorphism among Avena species revealed by retrotransposon-based DNA markers and soluble carbohydrates in seeds
Source: J Appl Genet. 2023 Jan 31;64(2):247–64. doi: 10.1007/s13353-023-00748-w (PMC10076396; doi:10.1007/s13353-023-00748-w)
Supplement: Supplementary file 6 — List of the Avena accessions used in the study. Species arranged alphabetically. (DOCX 18 kb) [file 13353_2023_748_MOESM4_ESM.docx]

**Table S1.** List of the *Avena* accessions used in the study. Species arranged alphabetically.

| **Species** | **Accession** | **Acquisition source** | **ASC** |
| --- | --- | --- | --- |
| *A. abyssinica* | 50633 | NCPGR | * |
| *A. abyssinica* | 51193 | NCPGR | * |
| *A. abyssinica* | K-14813 | VIR | * |
| *A. abyssinica* | K-14815 | VIR | * |
| *A. abyssinica* | NGB 6368 | NGB | * |
| *A. abyssinica* | NGB 8767 | NGB | * |
| *A. atlantica* | 51862 | NCPGR | * |
| *A. atlantica* | 51863 | NCPGR | * |
| *A. barbata* | 50755 | NCPGR | * |
| *A. barbata* | 52057 | NCPGR | * |
| *A. barbata* | NGB 9221 | NGB | * |
| *A. barbata* | 9222 | NGB | * |
| *A. byzantina* | 50074 | NCPGR |  |
| *A. byzantina* | 50133 | NCPGR | * |
| *A. byzantina* | 50218 | NCPGR | * |
| *A. byzantina* | 50402 | NCPGR |  |
| *A. byzantina* | 50696 | NCPGR |  |
| *A. byzantina* | K-14395 | VIR | * |
| *A. byzantina* | K-14916 | VIR | * |
| *A. byzantina* | NGB 5131 | NGB | * |
| *A. byzantina* | NGB 9814 | NGB | * |
| *A. damascena* | 51820 | NCPGR | * |
| *A. damascena* | 51821 | NCPGR | * |
| *A. damascena* | 51835 | NCPGR |  |
| *A. damascena* | 51846 | NCPGR |  |
| *A. damascena* | 51850 | NCPGR |  |
| *A. fatua* | 51608 | NCPGR | * |
| *A. fatua* | 51633 | NCPGR | * |
| *A. fatua* | 51635 | NCPGR | * |
| *A. fatua* | 51636 | NCPGR |  |
| *A. fatua* | 52149 | NCPGR |  |
| *A. hirtula* | 51847 | NCPGR | * |
| *A. hirtula* | 52150 | NCPGR | * |
| *A. hirtula* | 52346 | NCPGR | * |
| *A. insularis* | 52436 | NCPGR | * |
| *A. insularis* | 52438 | NCPGR | * |
| *A. longiglumis* | 52443 | NCPGR | * |
| *A. nuda* | 51277 | NCPGR | * |
| *A. nuda* | NGB 6370 | NGB | * |
| *A. nuda* | NGB 6371 | NGB | * |
| *A. nuda* | NGB 6369.1 | NGB | * |
| *A. sativa* | 50338 | NCPGR | * |
| *A. sativa* | 51521 | NCPGR |  |
| *A. sativa* | 52267 | NCPGR | * |
| *A. sativa* | 52433 | NCPGR | * |
| *A. sativa* | K-14717 | VIR | * |
| *A. sativa* | K-14784 | VIR | * |
| *A. sterilis* | 51555 | NCPGR |  |
| *A. sterilis* | 51557 | NCPGR | * |
| *A. sterilis* | 51848 | NCPGR |  |
| *A. sterilis* | 51851 | NCPGR | * |
| *A. sterilis* | NGB 11089.1 | NGB | * |
| *A. sterilis* | NGB 9219 | NGB | * |
| *A. sterilis* | NGB 9220 | NGB | * |
| *A. strigosa* | 502858 | NCPGR | * |
| *A. strigosa* | 51613 | NCPGR |  |
| *A. strigosa* | 51754 | NCPGR | * |
| *A. strigosa* | K-14943 | VIR | * |
| *A. strigosa* | K-15024 | VIR | * |
| *A. strigosa* | 51582 | NCPGR |  |

NCPGR - National Centre for Plant Genetic Resources;

NGB - Nordic Gene Bank (NGB);

VIR - N.I. Vavilov All-Russian Institute of Plant Genetic Resources (VIR);

ASC – accessions selected for analysis of soluble carbohydrates profiles
